# The quest for the best: manual, atlas- and spatial prior-based delineation of locus coeruleus

**DOI:** 10.1016/j.nicl.2026.104039

**Published:** 2026-07-21

**Authors:** Olga Dmitrichenko, Giuseppina Baldizzi, Tatjana Schmidt, Aurélie Bussy, Olivier Colliot, Antoine Lutti, Ferath Kherif, Bogdan Draganski

**Affiliations:** a Imaging Neuroscience of Ageing – inAGE laboratory, Neurology Department - Inselspital, University Hospital Bern, University Bern, Bern, Switzerland; b University Institute for Diagnostic and Interventional Neuroradiology - Inselspital, University Hospital Bern, University Bern, Bern, Switzerland; c Graduate School for Health Sciences, University of Bern, Switzerland; d Laboratory for Neuroimaging Research, Department of Clinical Neuroscience, Lausanne University Hospital and University of Lausanne, Lausanne, Switzerland; e Department of Clinical Neurosciences, University of Cambridge, Cambridge, Biomedical Campus, UK; f Sorbonne Université, Institut du Cerveau – Paris Brain Institute - ICM, CNRS, Inria, Inserm, AP-HP, Hôpital de la Pitié-Salpêtrière, Paris, France

**Keywords:** atlas, locus coeruleus, brainstem nuclei, neuromelanin MRI, quantitative MRI, relaxometry

## Abstract

Despite advances in neuromelanin-sensitive brain imaging and a plethora of software solutions, the reliable non-invasive delineation of the locus coeruleus (LC) in the human brainstem remains challenging. We sought to evaluate the spatial accuracy and consistency of atlas- and probabilistic spatial prior-based LC delineation. We acquired neuromelanin-sensitive 3 T MRI data in healthy volunteers (*n* = 24; mean age 40.0 ± 16.8 years; 42% female). Manual labelling by 9 raters performed twice provided the basis for individual- and group-level comparisons, showing moderate inter-rater agreement (mean Dice = 0.7). For the atlas-based labelling, we tested seven open-access LC atlases, a consensus reference representing the atlases' overlap and the averaged manual labelling. Open-access atlases demonstrated low spatial concordance (Dice = 0.2–0.4), while the averaged manual labelling atlas had higher spatial overlap (Dice = 0.6). Probabilistic delineation using spatial priors showed the strongest voxel-wise similarity with manual labelling (*r* = 0.3) when the averaged manual labelling atlas was used as prior. Principal component analysis confirmed the greater spatial compactness for atlas-based labelling. Atlas-based labelling using the averaged manual labelling atlas in an extended 3 T cohort (*n* = 2393, mean age 58.44 ± 13.75 years) identified that tissue myelin and iron declined continuously from early adulthood, while free tissue water increased – a neurobiological trend robust to atlas choice. LC volume showed an inverted-U trajectory. Our results highlight the potential of atlas-based labelling for LC identification and demonstrate its sensitivity to physiologically grounded aging processes, suggesting that harmonised validation strategies and context-sensitive approaches can improve reliability.

## Highlights

• Manual locus coeruleus labelling by 9 raters shows moderate inter-rater agreement.
• Atlas-based labeling offers higher spatial consistency than LC prior-based methods.
• Large-scale utility: Atlas methods capture age-related LC changes in 2300+ adults.
• LC volume shows a non-linear, inverted-U path, declining only after the mid-50s.
• Tissue microstructure differences precede LC volume loss across the lifespan.

## 1 Introduction

The locus coeruleus (LC), known as the “blue spot” due to its high neuromelanin content, is a small brainstem nucleus measuring approximately 15 mm rostro-caudally and 2.5 mm medio-laterally (Baker et al., 1989; Fernandes et al., 2012; Mouton et al., 1994; Pamphlett et al., 2018, Pamphlett et al., 2020). LC's critical involvement in neurodegenerative disorders explains the plenitude of studies that bring empirical evidence for disease-related anatomical differences (Lakhani et al., 2024; Trujillo et al., 2024; Zecca et al., 2002; Zecca, Youdim, et al., 2004). Consequently, there is a growing interest in establishing LC-centred imaging biomarkers, sensitive to early presymptomatic stages in neurodegeneration and especially in Alzheimer's disease (Braak et al., 2011; Satoh and Iijima, 2019). Beyond neurodegeneration, there is expanding clinical interest in mapping LC structural and functional characteristics across a broader spectrum of neuropsychiatric and functional neurological disorders, where altered LC-driven arousal dynamics and network transitions are increasingly implicated in clinical symptoms like functional seizures (Myren et al., 2026).

Unlike the basal ganglia, LC noradrenergic neurons feature relatively low iron and high copper content (Zecca, Stroppolo, et al., 2004), which makes it challenging to detect in vivo using conventional iron-sensitive magnetic resonance imaging (MRI). Dedicated neuromelanin-sensitive MRI (NM-MRI) protocols improve LC visualization by leveraging the specific paramagnetic properties of the melanin‑iron complex inside neuromelanin (Enochs et al., 1997; Keren et al., 2015; Li et al., 2022) that correlate with post-mortem LC localization (Sasaki et al., 2006). NM-MRI has prompted a line of research on LC delineation using the absolute and relative signal intensity characteristics, or combined with LC-specific spatial priors (Flores et al., 2025) for inferences about disease-specific effects (Lee et al., 2024). This renders NM-MRIs well-suited for manual labelling of the LC in small-sized group studies (Clewett et al., 2016; Mather and Harley, 2016), but their relative applicability and accuracy against atlas-based detection methods in large-scale cohorts using 3 T clinical scanners warrant further evaluation.

Atlas-based labelling of the LC represents a widely used alternative to the manual delineation. However, the accuracy of atlas-led LC definition largely depends on the atlas representativeness with reference to demographics, sample recruitment and sample size characteristics (Lee et al., 2024; Manaye et al., 1995). This could explain the modest spatial overlap of several freely available LC atlases (Dahl et al., 2019; Liu et al., 2019; Tona et al., 2017; Ye et al., 2021). Another source of potential delineation accuracy loss is the spatial registration precision between standard and individuals' native space. The anisotropic spatial resolution of the majority of NM-MRI protocols, the reduced field-of-view acquisition, and inferior–superior positioning rather than alignment to the anterior commissure–posterior commissure line, covering only the brainstem, introduce additional challenges for spatial registration (Trujillo et al., 2024). Transforming individual LC atlases into standard space typically results in moderate cross-subject overlap (<62%), with variability further influenced by image quality and sample characteristics (Yi et al., 2023). This is supported by empirical evidence showing that smaller structures are disproportionally more impacted by the choice of the applied linear and non-linear interpolations of spatial registration (Ashburner and Friston, 2011; Avants et al., 2008; Klein et al., 2009). Advances in diffeomorphic registration algorithms focusing on the brainstem carry the promise of mitigating this issue (Ashburner, 2007; Avants et al., 2008; Betts et al., 2017; Fonov et al., 2011).

Computational anatomy techniques using MRI and evolving feature extraction algorithms overcome the limitations of manual and atlas-based labelling methods (Ashburner et al., 2003). The recent developments in atlas-guided or machine-learning-driven feature extraction (Aganj et al., 2024; Dünnwald et al., 2021) offer additional benefits in terms of time efficiency and consistency. However, the LC's fusiform elongated shape and anatomical location make the robust and reliable delineation difficult (Liu et al., 2017; Yi et al., 2023). Automated approaches such as the funnel-tip method has been successfully benchmarked against manual labelling (intraclass correlation coefficient = 0.91) (Sibahi et al., 2023).

Considering the lack of a non-invasive in vivo imaging technique that provides the ground truth for LC spatial extent, the existing literature encompasses a wide range of arbitrarily chosen methods for validating the reported delineation accuracy. To address this challenge, we provide empirical evidence to guide informed decisions regarding LC atlas selection by quantifying spatial consistency and alignment accuracy. Here, we systematically evaluated three commonly used strategies for LC delineation: manual labelling, atlas-based, and LC-specific spatial prior–based delineation. We use multi-contrast MRI acquisitions with repeated annotations from several expert raters and cross-atlas comparisons to disentangle methodological from anatomical sources of variation. While deep-learning approaches have recently shown promise for automated LC segmentation, they generally rely on large, heterogeneous datasets and can be difficult to interpret in terms of anatomical details. To demonstrate the utility of the atlas-based approach, we tested the derived LC regions in a secondary analysis of aging trajectories in an extended cohort. Using quantitative MRI (qMRI) maps sensitive to myelin, iron, and tissue water content (Lorio et al., 2016), we aimed to determine whether atlas-based delineation could capture the hypothesized temporal dissociation between microstructural changes and volume loss (Callaghan et al., 2015; Manaye et al., 1995; Theofilas et al., 2017). We propose a complementary, study-specific approach to build a consensus LC atlas within established frameworks that capture inter-individual variability. The consensus atlas can then be used for future methods (including deep learning) and clinical studies.

## 2 Methods

### 2.1 Participants

For this study, we recruited 26 healthy volunteers. Two participants were excluded from further analysis because of inconsistent NM-MRI protocol parameters, resulting in a final sample of 24 individuals (10 females, 14 males; mean age 40.0 ± 16.8 years). The study was approved by the institutional Ethics Committee, and informed written consent was obtained from all participants (CER-VD, project number PB_2018–00038 (239/09)).

### 2.2 MRI data acquisition

All MRI data were acquired on a 3 T whole-body system (Prisma, Siemens Healthcare, Erlangen Germany) with a standard 32-channel radiofrequency receive head coil and body coil for transmission.

We used a T1-weighted (T1w) magnetization-prepared rapid acquisition gradient echo (MPRAGE) 3D gradient-recalled inversion recovery (GR-IR) protocol with the following parameters: repetition time (TR) = 2000 ms, echo time (TE) = 2.39 ms, inversion time (TI) = 920 ms, and flip angle α = 9° yielding isotropic voxel sizes of 1 × 1 × 1 mm.

The neuromelanin-sensitive protocol consisted of 2D gradient-echo (GRE) acquisitions in transverse orientation with enabled magnetization transfer contrast, optimized for neuromelanin with TR = 274 ms, TE = 2.28 ms, flip angle α = 40°, voxel size = 0.51 × 0.51 × 3.24 mm, and field of view (FoV) = 180 mm providing 12 interleaved slices with five signal averages.

To evaluate normative aging trajectories of the LC, we used a quantitative multiparametric mapping (MPM) protocol. This included three multi-echo 3D fast low-angle shot (FLASH) acquisitions with magnetization transfer-weighted (MTw: TR = 24.5 ms, α = 6°), proton density-weighted (PDw: TR = 24.5 ms, α = 6°), and T1-weighted (T1w: TR = 24.5 ms, α = 21°) contrasts at 1 mm isotropic resolution (Draganski et al., 2011; Weiskopf et al., 2013). To account for RF transmit field inhomogeneities (Lutti et al., 2014), mapping data were acquired using a 3D EPI spin-echo and stimulated echo method (Lutti et al., 2010, Lutti et al., 2012) (4 mm^3^ resolution, TE = 39.06 ms, TR = 500 ms). B0-field mapping was acquired to correct for image distortions in the EPI data (2D double-echo FLASH, TR = 1020 ms, TE1/TE2 = 10/12.46 ms, α = 90°). The total acquisition time for the MPM protocol was 27 min.

### 2.3 Unified approach and spatial registration estimates

We evaluated complementary approaches for LC delineation: atlas-based labelling and SPM12's “unified segmentation” (Wellcome Centre for Human Neuroimaging, University College London, UK) augmented with a LC-specific tissue prior whilst comparing with manual labelling by trained raters. For each approach, analyses were repeated across several freely available LC atlases to independently assess the effects of atlas choice versus delineation method.

**Fig. 1:**
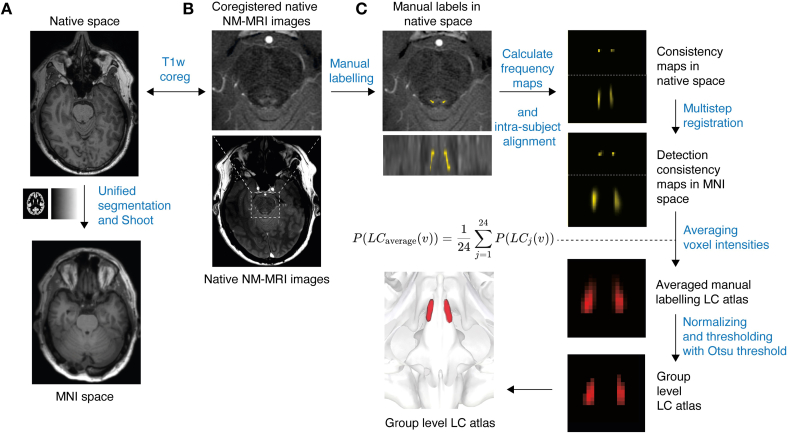
**A.** T1w image segmentation and spatial registration to MNI space. **B.** NM-MRI co-registered and resampled to isotropic resolution. **C.** Manual labels in native space, summed up to frequency maps and registered to MNI space. Averaged frequency maps thresholding to create a manual labelling LC atlas. *Abbreviations: T1w = T1-weighted; MNI = Montreal Neurological Institute; NM-MRI = neuromelanin-sensitive magnetic resonance imaging; LC = locus coeruleus.*

Aiming to evaluate the different methods' output in both native and standard Montreal Neurological Institute (MNI) space, we estimated individuals' spatial registration parameters using whole-brain T1w data, which provided the deformation fields and served as anatomical reference to anchor the reduced field-of-view of NM-MRI scans. To this end, we applied SPM12's segmentation with enhanced tissue priors (Lorio et al., 2016), followed by registration using the geodesic shooting algorithm. To ensure spatial consistency, we linearly registered the NM-MRI to the native space T1w images using 6-parameter rigid-body registration with nearest neighbor interpolation. While non-linear registration is generally preferred for ensuring optimal whole-brain alignment, a linear approach was applied here, given the specific LC spatial extent and location in the brainstem. This renders a linear alignment sufficient to account for the acquisition volume tilt and translation while remaining robust against the localized warping artifacts that non-linear deformations can inadvertently introduce for small structures such as the LC. During this step, we resampled the NM-MRI data from their original voxel size (0.51 × 0.51 × 3.24 mm) to an isotropic resolution of 1x1x1mm using nearest neighbor interpolation (full preprocessing in Fig. 1**, A-B).**

### 2.4 Manual labelling

The LC was manually labelled twice, in all three planes, on 24 NM-MRIs by a team of 9 independent raters ranging from PhD candidates to postdoctoral researchers from the lab, following instructions on software use and LC identification. To ensure spatial consistency and minimize inter-observer variability, all raters were trained by the same expert investigator using an identical neuroanatomical protocol for LC identification prior to dataset labelling. Raters were not blinded to subject identifiers but completed all first pass labelling prior to initiating the second round. For visualization and labelling, we used the MINC Toolkit (version 1.9.18, https://bic-mni.github.io).

We assessed the intra-rater reliability of manual labelling using the Dice-Sørensen coefficient (Dice) for each annotated voxel. The Dice was calculated as twice the number of voxels that were labelled in both annotations (from the 2 repetitions of the same rater), divided by the sum of the voxels labelled in each annotation. Inter-rater reliability was assessed using the Fleiss' Kappa index. Since the LC contains only a few voxels, we restricted the search volume to a region of interest (ROI) surrounding the expected LC location, defined by its coordinates in MNI space (x = 82–98, y = 80–100, z = 0–60) to reduce potential biases from non-LC voxels. The Fleiss' Kappa index was calculated on a voxel basis within the ROI across all raters and datasets.

We created detection frequency maps by accumulating labels at the voxel level across raters and repetitions, reflecting raw counts. These maps were then co-registered and resampled to 1 × 1 × 1 mm with trilinear interpolation, followed by warping in MNI space using the already estimated diffeomorphic spatial registration parameters. We generated a group-level atlas by averaging detection frequencies across subjects (from now on denoted as averaged manual labelling atlas or group-level atlas). Using Otsu's thresholding, we excluded low-agreement voxels and minimized alterations from the spatial registration in the averaged manual labelling atlas. The remaining values were either scaled to the [0,1] range for group-level atlas creation or set to 1 for a binary version (see Suppl. Fig. 1). At each step, we visually inspected image quality and spatial alignment (see Fig. 1**, C).**

### 2.5 Overview of included LC atlases

The main characteristics of the open-access LC atlases (Betts et al., 2019; Dahl et al., 2019; Keren et al., 2009; Lee et al., 2024; Liu et al., 2017; Tona et al., 2017; Ye et al., 2021) included in our analyses are summarised in Table 1.

**Table 1:** Overview of included LC atlases.

*Author*	Cohort / Source Study	Total Sample (*N*)	Age Range & Sub-Sample Breakdown	LC delineation	Variability	MRI field & scan resolution	Voxel size	Processing software	Atlas assessment	Spatial overlap
Betts et al., 2017	DZNE Longitudinal Cognitive Impairment and Dementia study	82	• Young: *n* = 25 (22–30 years) • Older: *n* = 57 (61–80 years)	Manual (1 rater)	Deterministic	3 T, T1w MPRAGE (1 × 1 × 1 mm), T1w FLASH (0.75 × 0.75 × 0.75 mm)	0.5 × 0.5 × 0.5 mm	ANTs v2.1, MATLAB R2014b	Median/max LC signal relative to rostral pontomesencephalic reference	No statistical comparison
Dahl et al., 2019	Berlin Aging Study-II)	294	• Young: *n* = 66 (25.4–39.8 years) • Older: *n* = 228 (60.2–80.4 years)	Semi-automated based on peak intensities	Probabilistic	3 T, T1w MPRAGE (1 × 1 × 1 mm), NM-MRI T1w TSE (0.5 × 0.5 × 0.5 mm)	0.5 × 0.5 × 0.5 mm	ANTs v2.1	Peak LC voxel intensities and LC-to-reference ratios per hemisphere	Betts 57.71%, Keren 1SD 69.14%
Keren et al., 2009	Population-based atlas	44	Full Range: *N* = 44 (19–79 years)	Intensity-based manual ROI	Deterministic (max voxel at 1–2 SD)	3 T, T1-TSE (0.4 × 0.4 mm in-plane; 3 mm slice thickness)	0.5 × 0.5 × 0.5 mm	FSL 4.0 FAST, MRIcro, SPM5	LC signal frequency in T1-TSE vs. postmortem cell counts	Cell counts vs. postmortem
Lee et al., 2024	Paediatric/Developmental cohort	66	Children/Adolescents: N = 66 (8–19 years)	Iterative nonlinear template-based segmentation	Probabilistic (majority voting, multi-threshold)	3 T, T1-FSE (0.43 × 0.43 mm in-plane; 2.5 mm slice thickness), T1w (1 × 1 × 1 mm), T2w (1 × 1 × 1 mm)	1 × 1 × 1 mm	FSL 6.0.1., ANTs	Iteration evaluation of mean differences: contrast, boundary definition, and variability	None
Liu et al., 2019	Cam-CAN project	605	Lifespan Range: *N* = 605 (18–88 years)	Manual (2 raters)	Deterministic	3 T, MTw SPGR (1.5 × 1.5 × 1.5 mm), T1w MPRAGE (1 × 1 × 1 mm)	0.5 × 0.5 × 0.5 mm	ANTs v2.1	Mean LC signal relative to pons reference region (left/right)	Dahl 94%, Keren 1SD 48%
Tona et al., 2017	Young adult cohort	17	Young Adults: *N* = 17 (19–24 years)	Manual (2 raters × 2 repetitions)	Probabilistic (spatial overlap, L/R range)	3 T, T1w TSE (0.87 × 0.87 × 1.2 mm whole brain; 0.35 × 0.35 × 1.5 mm brainstem)	0.5 × 0.5 × 0.5 mm	FSL 5.0.8.	Reliability via inter‐/intra-rater Dice, ANOVAs, ICC; LC volume across raters, sessions, hemispheres	Volume vs. postmortem (incl. Keren)
Ye et al., 2021	Aging Cohort	53	Older Adults: *N* = 53 (52–84 years)	Semi-automated based on volume and CNR estimation	Probabilistic	7 T, MT-TFL (0.4 × 0.4 × 0.5 mm), T1w MPRAGE (0.7 × 0.7 × 0.7 mm)	0.5 × 0.5 × 0.5 mm	ANTs v2.2.0, Matlab R2018b, SPM12	SNR in LC and reference ROIs (SNR/MTR, MT-on); mean intensities; asymmetry tested (*t*-tests)	Visual 3D (Keren, Tona, Betts)

Abbreviations: 3 T = 3 Tesla; 7 T = 7 Tesla; ANTs = Advanced Normalization Tools; CNR = Contrast-to-Noise Ratio; FSL = FMRIB Software Library; ICC = Intraclass Correlation Coefficient; MPRAGE = Magnetization-Prepared Rapid Gradient Echo; MTw = Magnetization Transfer weighted; MT-TFL = Magnetization Transfer Turbo Flash; NM-MRI T1w TSE = Neuromelanin-sensitive T1 weighted Turbo Spin Echo; ROI = Region of Interest; SD = Standard Deviation; SNR = Signal-to-Noise Ratio; SPGR = Spoiled Gradient Recalled; T1w = T1 weighted; T1-FSE = T1 weighted Fast Spin Echo; T1-TSE = T1 weighted Turbo Spin Echo; T2w = T2 weighted.

### 2.6 Atlas-based labelling

**Fig. 2:**
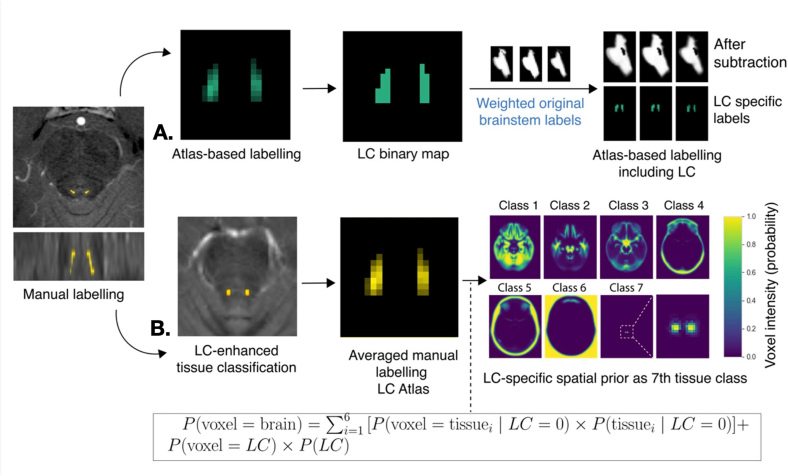
Manual LC labels from NM-MRIs used for: **A.** atlas-based labelling and **B.** SPM12's unified segmentation with LC-spatial prior. *Abbreviations: LC = locus coeruleus; NM-MRI = neuromelanin-sensitive MRI; SPM = Statistical Parametric Mapping; TPM = tissue probability map.*

To enable a fair comparison, we used two atlas sets: the freely available LC atlases and the group-level LC atlas generated by averaging manual labels (Suppl. Table 1B). For each atlas, we generated voxel-wise probabilistic and thresholded binary labels based on a maximum probability criterion. We integrated those labels alongside the 125 cortical and subcortical labels from the “MICCAI 2012 Grand Challenge and Workshop on Multi-Atlas Labeling” (https://my.vanderbilt.edu/masi/about-us/resources-data). The LC label was excluded from the brainstem label (see Fig. 2). To achieve this, we resampled the atlases to an isotropic voxel size of 1.5 × 1.5 × 1.5 mm for integration in the MICCAI label set. This spatial resampling inherently introduces voxel-grid mismatches; down-sampling the high-resolution atlas (e.g., 0.5 mm isotropic templates) requires interpolation that may dilute fine structural boundaries through partial volume effects, which can artificially expand or blur the nominal borders of a structure as tiny as the LC*.* The atlas-based labelling included spatial registration and labelling in individuals' native space (Ashburner and Friston, 2011).

Aiming to mitigate manual labelling variability, we created a consensus LC reference by combining information from the seven open-access atlases and with the averaged manual labelling LC atlas within the atlas-based labelling. The overlap maps quantified, per voxel, the number of atlas volumes in which each voxel was represented. Voxels present in two or more atlases were retained to define a consensus representation of the LC (Suppl. Fig. 2). This resulting consensus-based reference atlas was added as an additional atlas-based ROI, generating a harmonised benchmark output.

### 2.7 LC-enhanced tissue classification

We used SPM12's unified segmentation for automated tissue classification of the NM-MRIs, incorporating a LC-specific spatial prior as an extra tissue class and NM-MRI signal intensity modelled with two Gaussians (see Suppl. Table 1 A) in 1 × 1 × 1 mm resolution. To assess dependence on the prior, we repeated the procedure in three ways: with each of the seven already published atlases, with the averaged manual labelling atlas or with the consensus reference atlas described above.

### 2.8 Validation

The following features were assessed for both spatial priors-based and LC atlas-based labelling: (i) spatial overlap, (ii) anatomical alignment, (iii) intensity distributions and spatial patterns, and (iv) boundary distances. Spatial overlap was measured using: (i) soft Dice, calculated by taking twice the voxel-wise product of the two probabilistic outputs, summing over all voxels, and dividing by the sum of the squared voxel values of each output, providing a continuous generalisation of the standard Dice for non-binary labels; (ii) volume intersection, which is the ratio of intersecting voxels to the union of voxels in two outputs, capturing agreement in overall volume and (iii) voxel-wise Pearson correlation, measuring the correlation between corresponding voxel values. Anatomical alignment was assessed using the Euclidean distance between the centres of mass (COM). Intensity distributions and spatial patterns were compared using the Kullback–Leibler (KL) divergence and Earth Mover's Distance (EMD), which determine how much and how far intensity values must be shifted to transform one spatial pattern into another without simplifying the data into binary masks. Boundary distances and outliers were quantified through the Hausdorff Distance.

We assessed the performance of the automated LC delineation approaches by measuring their spatial overlap with the corresponding consensus reference. This comparison was done in both native and MNI space, focusing specifically on the rostro-caudal dimension. To account for the small sample size and non-normal distribution of these metrics and provide robust estimates of uncertainty, we used a non-parametric bootstrapping approach. For each metric and atlas, the sample mean and 95% confidence intervals (CIs) were estimated by resampling the subject data with replacement over 2000 iterations.

We analysed the anatomical distribution of the LC-spatial prior-based and atlas-based outputs by comparing the rostro-caudal (z-axis) and in-plane (x- and y- axis) spatial extent, considering the impact of the necessary arbitrary thresholding of the probabilistic LC maps. We used principal component analysis (PCA) on the voxel coordinates to attribute the density distributions of voxel signal along each spatial component, which we interpreted as corresponding to the anterior–posterior (PC1), left–right (PC2), and inferior–superior (PC3) axes of LC. We compared spatial dispersion across methods and used group-level statistics to test for significant differences. Analyses were conducted using NumPy and pandas for data manipulation, with visualization performed via seaborn and matplotlib and statistical testing supported by *SciPy* and *statsmodels*.

### 2.9 Aging trajectories of the LC

For the analysis within the predefined atlas-based ROI, we used an extended study sample of participants in the BrainLaus project, a nested study of the epidemiological CoLaus|PsyCoLaus longitudinal cohort (*n* = 2393; mean age 58.44 ± 13.75 years; range 25–80 years). The BrainLaus MRI protocol comprises multi-shell diffusion-weighted, T1-weighted and task-based functional MRI acquisitions (Ramponi et al., 2026), in addition to relaxometry quantitative MRI (qMRI). All MRI data were acquired on the same MRI machine, the same 64-channel head coil, the same customized acquisition sequences, and an unchanged scanner software version throughout the study period. qMRI maps were calculated using the voxel-based quantification (VBQ) approach implemented in the hMRI toolbox (Draganski et al., 2011; Tabelow et al., 2019).

The relaxometry protocol (Draganski et al., 2011), allows for calculating maps of magnetization transfer saturation (MTsat), transverse relaxation rate (R2*), longitudinal relaxation rate (R1) and effective proton density (PD*) considered as markers of myelin (Callaghan et al., 2014; Lutti et al., 2014; Stanisz et al., 1999), iron (Fukunaga et al., 2010; Kirilina et al., 2020; Stüber et al., 2014) and unbound water (Lin et al., 1997; Watanabe et al., 2019) content (Edwards et al., 2018; Weiskopf et al., 2015). The BrainLaus qMRI data has been used to characterise brain microstructure aging trajectories and associated lifetime factors (Draganski et al., 2011; Slater et al., 2019; Taubert et al., 2020; Trofimova et al., 2021, Trofimova et al., 2023), white-matter hyperintensities (Bussy et al., 2026) and microstructural covariance in brain disorders (Accolla et al., 2014; Melie-Garcia et al., 2017; Santos et al., 2023; Weitnauer et al., 2021).

## 3 Results

### 3.1 Atlas-based labelling performance

#### 3.1.1 Manual label validation and group-level atlas construction

**Fig. 3:**
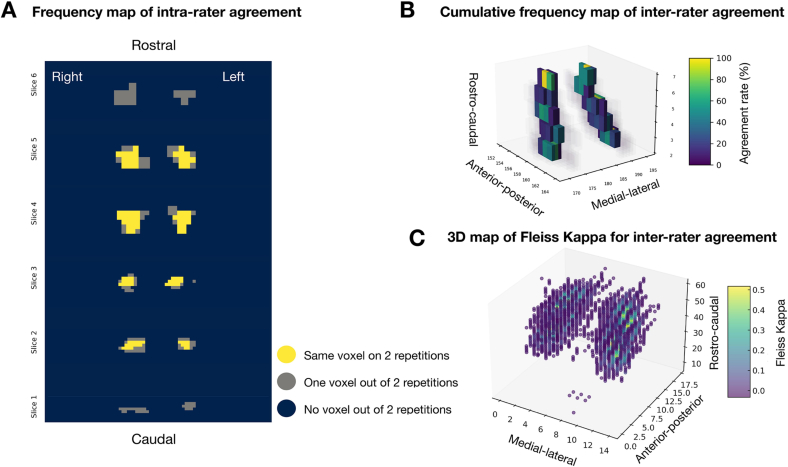
**A.** Example of intra-rater agreement maps across six rostro-caudal slices by one rater: yellow = consistent voxels, grey = single repetition, dark blue = no overlap. **B.** Cumulative frequency map of inter-rater agreement (0–100%). Colour intensity indicates relative frequency values. **C.** 3D Fleiss' Kappa map (0–0.5) in MNI space of inter-rater reliability along rostro–caudal, anterior–posterior, and medio–lateral axes across all raters and subjects. *Abbreviation: MNI = Montreal Neurological Institute.*

**Fig. 4:**
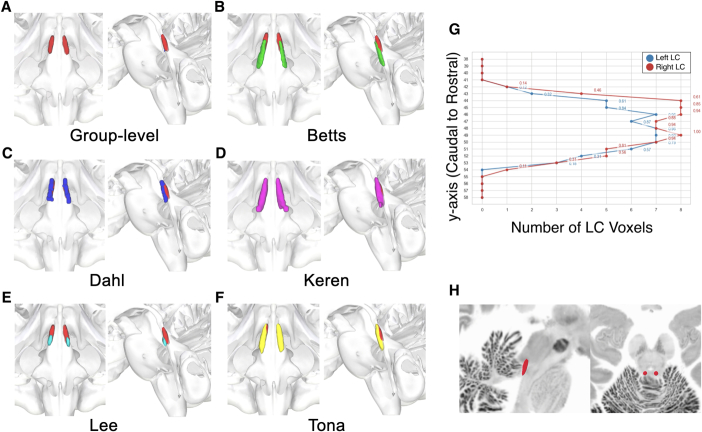
**A-F.** Renderings of LC atlases (group-level, Betts, Dahl, Keren, Lee, and Tona) in MNI space. **G.** Number of voxels in the left (blue) and right (red) group-level LC atlas along the rostro-caudal axis. **H.** Group-level atlas projected on the BigBrain. *Abbreviations: group-level = averaged manual labelling atlas; LC = locus coeruleus; MNI = Montreal Neurological Institute.*

The average Dice for the manual labelling across nine raters was 0.69 ± 0.18 (mean ± SD); 95% CI: 0.67–0.72, reflecting moderate intra-rater spatial agreement. The inter-rater agreement was also moderate, with a maximum Fleiss Kappa value of 0.5 (Fig. 3). The averaged manual labelling LC atlas (Fig. 4**)** extends rostro-caudally to 12 mm in MNI space. To test the stability of the atlas creation, we performed random sub-sampling validation by creating atlases from different subsets of 21 individuals and excluding randomly 3 individuals. The resulting atlases showed near-perfect overlap (>99%), indicating that the group atlas construction is highly stable and mathematically consistent regardless of the specific participant subset selected from our cohort.

#### 3.1.2 Native space delineation accuracy across open-access atlases

**Fig. 5:**
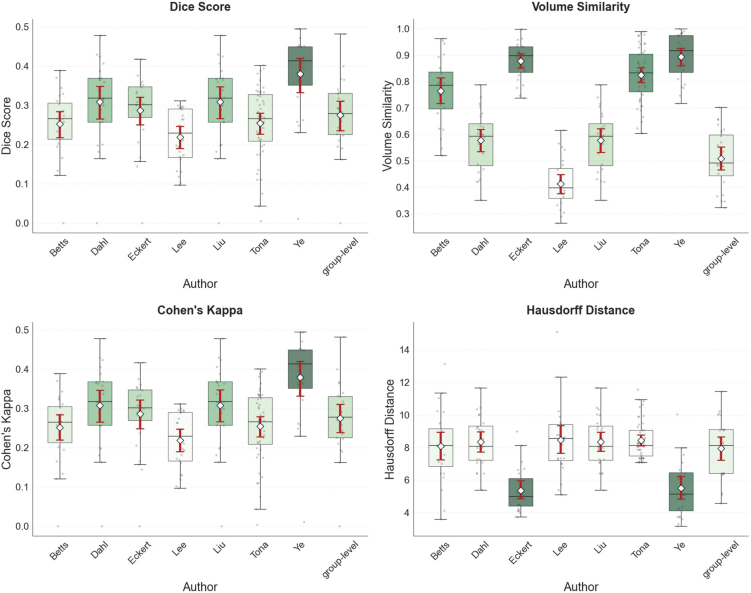
Performance comparison of atlas labelling against manual reference. Boxplots with median, interquartile ranges, and outliers. Colour intensity – degree of agreement with the reference manual label across all metrics. Hausdorff Distance – lower values correspond to darker shades, white diamonds – sample mean. Red error bars – 95% confidence interval of the mean, calculated via non-parametric bootstrapping (*n* = 2000 iterations). Individual subject data points overlaid as grey circles to illustrate distribution density. *Abbreviations: group-level = averaged manual labelling atlas.*

We integrated the LC atlases into the MICCAI multi-atlas framework. To evaluate the accuracy of the atlas-based labelling, we calculated the Dice between the individual atlas and manual label for each subject. The labelling accuracy varied across the different evaluated atlases (Fig. 5). Dice ranged from low values for the Lee atlas (0.22 ± 0.07, 95% CI 0.03) to the highest for the Ye atlas (0.38 ± 0.11, 95% CI 0.04), which showed a few outliers likely due to registration errors or anatomical variability. The Tona atlas exhibited high overlap (0.25 ± 0.09, 95% CI 0.03), while the averaged manual labelling atlas had lower overlap (group-level Dice = 0.28 ± 0.09, 95% CI 0.04). Cohen's Kappa values demonstrated a similar pattern across atlases and individuals. Volume intersection was highest for the Tona and Keren atlases (0.83 ± 0.10, 95% CI 0.03), and lowest for Lee (0.41 ± 0.09, 95% CI 0.04) and the group-level atlas (0.51 ± 0.11, 95% CI 0.04). Boundary distance measures (COM and Hausdorff) indicated that the Keren atlas produced the most consistent outputs, with the smallest distances to manual labels (Suppl. Table 2**)**.

#### 3.1.3 Spatial concordance against consensus reference

The analysis comparing atlas-based labels to the consensus reference (Suppl. Fig. 3) showed that the averaged manual labelling atlas had the highest spatial concordance (mean Dice coefficient = 0.63 ± 0.02, 95% CI 0.01), while the Tona and Liu atlases had the lowest. Volume similarity was generally high across most atlases, with the Lee atlas at the lowest performance range. Labels derived from the Ye and Keren atlases achieved the closest boundary distances to the consensus reference atlas, whereas the Tona atlas showed the greatest differences.

### 3.2 LC-specific spatial prior based delineation

#### 3.2.1 Tissue classification performance in native space

**Fig. 6:**
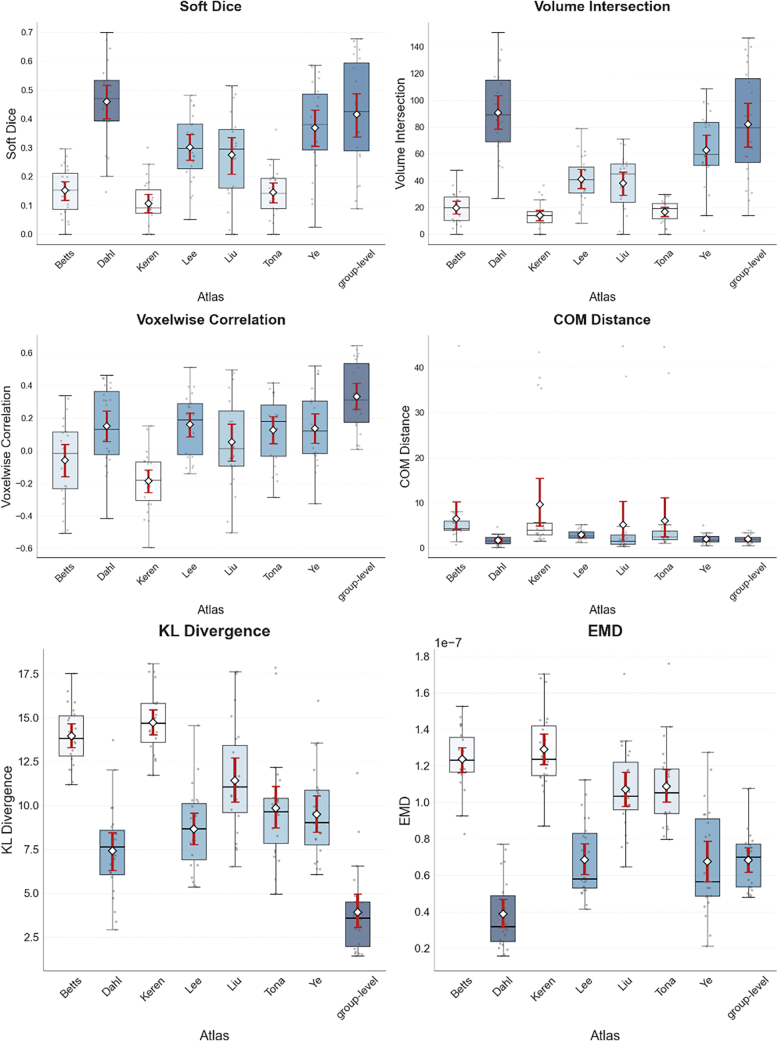
Comparison of LC-specific spatial prior-based delineation against manual labels in native space. Boxplots – median, interquartile ranges, outliers and individual data points. Colour intensity – degree of agreement with the reference manual label, white diamonds – sample mean. Red error bars – 95% confidence interval (CI) of the mean, derived from non-parametric bootstrapping (n = 2000 iterations). Individual subject metrics – grey circles showing the data distribution across the cohort. *Abbreviations: COM = centre of mass; EMD = Earth mover's distance; group-level = averaged manual labelling atlas; KL divergence = Kullback–Leibler divergence.*

In SPM12's tissue classification with a LC-specific prior using the group-level and Dahl atlas showed the most accurate and spatially consistent performance relative to manual labels in native space (Fig. 6). Using the Dahl atlas as a prior, we obtained the highest mean Soft Dice coefficient (0.46 ± 0.14, 95% CI 0.06) and volume intersection with the manual labels (90.57 ± 31.31, 95% CI 12.80). In contrast, using the group-level atlas as LC prior achieved the highest voxel-wise correlation (0.33 ± 0.21, 95% CI 0.08) and the lowest mean KL divergence (3.91 ± 2.47, 95% CI 1.01), indicating better overall spatial consistency. The COM distance analyses showed outliers in four out of the eight priors, with the Betts atlas-based prior showing the greatest variability (6.47 ± 8.58, 95% CI 3.50). Evaluation of KL divergence showed the group-level LC prior achieved the lowest mean divergence (3.91 ± 2.47, 95% CI 1.01). EMD was lowest for Dahl- and Ye atlas-based priors (Dahl atlas: 3.88E-08 ± 1.88E-08, 95% CI 7.67E-09; Ye: 6.77E-08 ± 2.86E-08, 95% CI 1.17E-08), though the Ye atlas based prior showed greater variability across subjects (for details see Suppl. Table 3).

#### 3.2.2 Prior delineation compared against consensus reference

We compared the created LC maps from the automated tissue classification against the consensus reference (Suppl. Fig. 4). The performance trends across authors observed for the Soft Dice and Volume Intersection remained consistent with those obtained from comparisons to manual labelling. Voxel-based correlation values decreased for all outputs in this comparison, except when using the group-level atlas as LC prior, which maintained higher correlation levels. The COM distance demonstrated a broader distribution of values compared to manual labelling assessments.

### 3.3 Comparative performance of LC atlases across delineation frameworks

The seven open-access LC atlases revealed distinct baseline variations in native voxel resolution, target voxel size and cohort demographics, which directly influenced final delineation performance depending on the chosen processing framework. When evaluated within the automated labelling framework where labels were downsampled to a uniform 1.5 × 1.5 × 1.5 mm resolution and binarized using Otsu thresholding, the 7 T-derived Ye atlas performed best. It showed the highest native-space spatial overlap (mean Dice = 0.38 ± 0.11; Volume Similarity = 0.89 ± 0.09). Conversely, the paediatric-based Lee atlas performed the poorest under these constraints (mean Dice = 0.22 ± 0.07; Volume Similarity = 0.41 ± 0.09). A similar pattern emerged for spatial boundary errors; the Keren and Ye atlases minimized boundary deviations, with mean Hausdorff Distances of 5.36 ± 1.36 mm and 5.52 ± 1.76 mm, respectively, whereas the Lee, Tona, Dahl, and Liu atlases clustered at higher boundary distances exceeding 8 mm.

This performance hierarchy shifted notably when the atlases were instead evaluated as localized spatial priors within SPM12s “unified segmentation” framework, resampled to a uniform 1 × 1 × 1 mm resolution with an optimized intensity threshold. In this probabilistic setting, the Dahl lifespan atlas achieved the highest spatial concordance against native space manual labels (mean Soft Dice score = 0.46 ± 0.14; Volume Intersection = 90.57 ± 31.31; center-of-mass (COM) Distance = 1.67 ± 1.08 mm). The Ye atlas followed (Soft Dice = 0.37 ± 0.16); Volume Intersection = 62.64 ± 28.92). In contrast, atlases with narrow or restricted paediatric age ranges showed decreased spatial capture; the young-adult Tona atlas yielded a mean Soft Dice of 0.14 ± 0.09 (Volume Intersection: 16.78 ± 9.12), while the paediatric Lee atlas reached a Soft Dice of 0.30 ± 0.12 (Volume Intersection: 41.09 ± 17.17). The lowest spatial prior overlap metrics overall were observed for the older, low-resolution Keren atlas (mean Soft Dice = 0.11 ± 0.08; mean COM Distance = 9.63 ± 13.53 mm).

Taken together, these results demonstrate that atlas influence depends on both the methodological approach and demographic alignment. High-resolution structural features, as in the 7 T-derived atlas, provided an accuracy advantage in atlas-based labelling. When atlases instead function as probabilistic spatial priors, broad lifespan representation, exemplified by the Dahl atlas, becomes the primary driver of performance. Paediatric cohorts and low-resolution atlases, by contrast, reduced delineation accuracy across both methodologies.

### 3.4 Spatial distribution and concordance of LC delineation methods

#### 3.4.1 Cross-sectional geometry

**Fig. 7:**
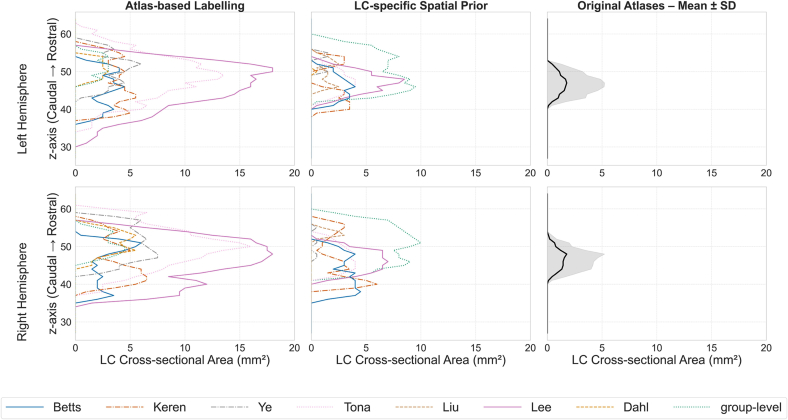
Cross-sectional LC area along the rostro–caudal (z-) axis in MNI space. *Columns 1–2*: per-atlas cross-sectional area profiles for atlas-based and spatial prior-based labelling (individual masks as separate lines). *Column 3*: original LC atlases summarised as mean ± 1 SD cross-sectional area (black line, grey shading). *Abbreviations: group-level averaged manual labelling atlas; LC = locus coeruleus; SD = Standard Deviation.*

To quantify inter-method consistency, spatially aligned outputs of atlas-based labelling and LC-specific spatial prior delineation were compared with respect to cross-sectional area along the rostro–caudal axis (Fig. 7). Both methods localized the structure within a comparable rostro–caudal extent, though the atlas-based labelling generally exhibited greater cross-sectional area across slices and hemispheres. The third column summarizes the eight LC atlases, depicting the mean cross-sectional area (lines) and ± 1 SD variability (shaded) as a visual reference for the atlas inputs and their transformation in the delineation outputs.

#### 3.4.2 Zonal reliability across tiers (standard vs. native space)

Given the variation in spatial overlap between delineation approaches, particularly along the rostro–caudal LC axis, we next evaluated their reliability in localizing the LC. The atlas-based labelling showed generally higher agreement in standard space, particularly for the Lee, Tona and Ye atlases, which achieved near-perfect overlap (>95%) across all three rostro–caudal LC tiers (see Suppl. Figs. 5 and 6). On the contrary, the overlap was consistently lower and more variable across atlases in native space, with especially low agreement in the rostral section (Tier 1) when dividing the LC in three tiers (Suppl. Fig. 7). Collapsing the LC to 2 tiers slightly improved average overlaps but did not resolve the spatial inconsistencies (Suppl. Fig. 8).

The LC delineation based on spatial priors showed lower overlap with the consensus reference (Suppl. Figs. 9–12), particularly in native space, where alignment across tiers was poor. Only the group-level and the Lee-based LC prior demonstrated high spatial overlap in MNI space, with the group-level atlas approximating 90% in Tier 2. Reducing the search volume from three to two tiers improved consistency in some cases but did not substantially elevate overall agreement.

#### 3.4.3 Principal component analysis of spatial variance orientation

**Fig. 8:**
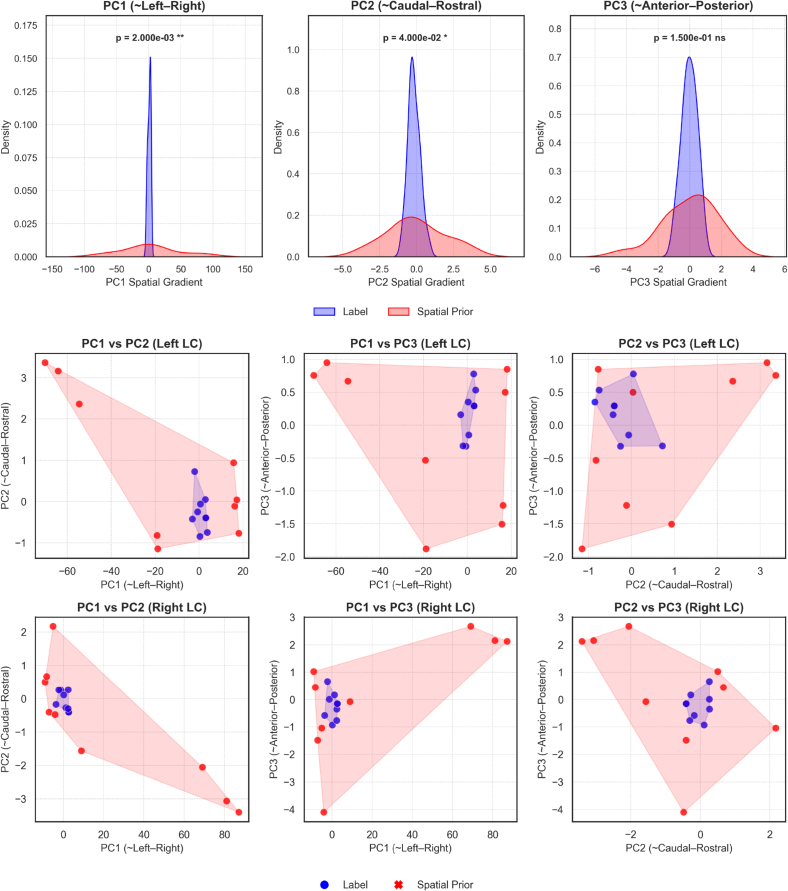
Voxel-wise spatial gradients across principal components (PCs) for atlas-based labelling (blue) and LC-spatial prior based tissue classification (red) in MNI space. Kernel density estimates for PC1 (left–right), PC2 (rostro-caudal), and PC3 (anterior–posterior). Scatter plots of paired PCs for left and right LC. Abbreviations: group-level = averaged manual labelling atlas; PC = Principal Component; ns = non-significant; * ≤0.05, ** ≤0.002.

Spatial organisation of detected LC voxels was summarised using PCA on atlas-based and LC-enhanced tissue classification outputs in MNI space. PCA revealed three major axes of spatial variance, approximately aligned with the anatomical left–right (PC1), caudal–rostral (PC2) and anterior–posterior (PC3) directions. The PCA component coordinates in MNI space were: PC1 = [0.914, 0.396, 0.091], PC2 = [0.098, −0.431, 0.897], PC3 = [−0.395, 0.811, 0.433]. The angular deviation between the PCA and AC–PC coordinate systems was [23.99°, 64.45°, 64.36°] for PC1, PC2, and PC3. The orientation of the PCA axes suggests that the LC is elongated along an oblique axis relative to the canonical AC–PC frame, in line with its anatomical rotation in the brainstem. Density plots for each principal component (Fig. 8**, top row**) showed spatial dispersion differences between approaches. The LC-specific spatial prior-based delineation showed broader voxel distributions across all components, particularly along the left–right (PC1) and caudal–rostral (PC2) axes, indicating a more spatially diffuse representation. In contrast, outputs of the atlas-based labelling were narrowly distributed and centred around zero, suggesting greater spatial coherence. The statistical testing confirmed significant differences between approaches along PC1 (*p* = 2.00 × 10^−3^, **), PC2 (*p* = 4.00 × 10^−2^, *), but not PC3 corresponding to the caudal–rostral direction (*p* = 1.50 × 10^−1^, ns). The voxel coordinate projections in PCA space (Fig. 8**, bottom row**) showed the differences in spatial delineation accuracy. The scatter plots confirm these patterns: atlas-based labelling clustered tightly in all planes, whereas spatial prior–based delineation showed greater dispersion, particularly along PC1 and PC2, indicating higher spatial variability.

### 3.5 Differential aging trajectories of the LC

#### 3.5.1 Contrast and relaxometry divergence (MTsat, R2*, R1, PD*)

**Fig. 9:**
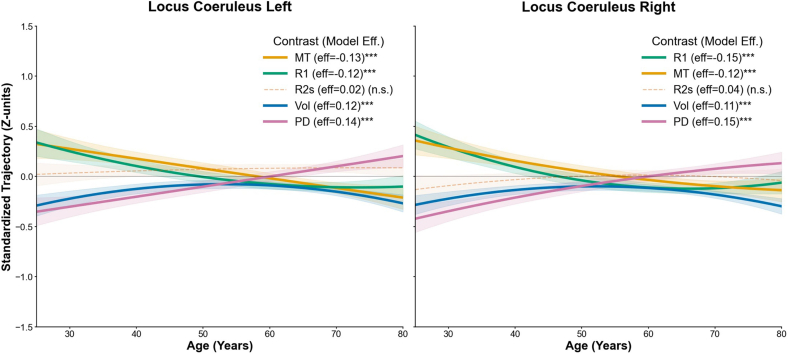
Aging trajectories of the locus coeruleus. Aging profiles relate standardized *Z*-scores from R1, R2*, MTsat, PD*, and volume maps to age (25–80 years) for the left and right LC. Predicted trajectories are derived from quadratic regression models (Age + Age^2^), with volume additionally corrected for total intracranial volume. Solid lines indicate significant age-related trajectories (P_FDR_ < 0.05) with 95% confidence intervals shaded; dashed lines indicate non-significant models. The legend is sorted by the model effect size (*eff*), while asterisks denote the significance of the overall quadratic model fit (∗*p* < 0.05, ∗∗*p* < 0.01, ∗∗∗*p* < 0.001). *Abbreviations: eff = model effect size; FDR = false discovery rate; MT = magnetization transfer saturation; n.s. = not significant; PD = proton density; R1 = longitudinal relaxation rate; R2s = transverse relaxation rate.*

Quadratic regression analysis revealed distinct aging profiles across MRI-derived quantitative maps for both the left and right LC. Significant associations were identified in both the timing (peak age) and the magnitude of standardized *Z*-score changes across the observed lifespan (25–80 years; Fig. 9). A temporal dissociation was observed between quantitative values and volume. The trajectory pattern was consistently observed across the evaluated atlases.

MTsat showed a continuous and pronounced decline from early adulthood (Left: P_FDR_ = 1.02 × 10^−9^, *eff* = −0.13; Right: P_FDR_ = 1.15 × 10^−8^, *eff* = −0.12), characterized by a monotonic linear-boundary trajectory and a total standardized reduction of −0.536 (Left) and − 0.495 (Right) by age 80.

Similarly, the longitudinal relaxation rate, R1, showed a significant age-related decrease (Left: P_FDR_ = 4.10 × 10^−8^, *eff* = −0.12; Right: P_FDR_ = 6.06 × 10^−9^, *eff* = −0.15), with trajectories reaching a late-life inflection point (Left: 71.9 years; Right: 66.0 years) and a total Z-drop of −0.458 and − 0.467, respectively. Conversely, PD* showed the most substantial deviation of all metrics, characterized by a continuous and highly significant lifespan increase (Left: +0.587 Z, *eff* = 0.14; Right: +0.571 Z, *eff* = 0.15; P_FDR_ < 6.6 × 10^−11^). In contrast, the effective transverse relaxation rate, R2*, did not show significant age-related changes in either hemisphere (Left: P_FDR_ = 0.561, *eff* = 0.02; Right: P_FDR_ = 0.416, *eff* = 0.04), with trajectories remaining near the cohort mean across the observed age range (see Suppl. Table 4).

While the magnitude of decline in MTsat was slightly more pronounced in the left LC, the directionality, morphology, and model effect sizes of all significant biophysical metrics were highly consistent across hemispheres.

#### 3.5.2 Non-linear volumetric trajectories

LC volume followed a non-linear quadratic path, maintaining structural stability until mid-adulthood before reaching a distinct inflection point at approximately 53.7 years (Left) and 53.6 years (Right). Following this peak, volumetric atrophy accelerated, resulting in a net Z-change of +0.062 and + 0.059 relative to the age-25 baseline (P_FDR_ < 7.2 × 10^−7^), reflecting an inverted-U trajectory. Notably, while linear Pearson correlations for volume were near-zero (see Suppl. Table 4), the quadratic model effect sizes (*eff* = 0.12 Left; 0.11 Right) revealed that age-related variance in volume is substantial when accounting for this non-linear morphology.

Aging trajectories demonstrated high hemispheric symmetry, particularly in volumetric measures, with left and right LC volumes reaching peak values within 0.1 years of each other.

## 4 Discussion

In this study, we compared atlas-based, and spatial prior-guided LC delineation against manual labelling of NM-MRI data, assessing spatial accuracy and consistency in native and standard spaces. Atlas-based labelling using group-level manual labels yielded the highest concordance with individuals' manual labels and the consensus reference, with lower spatial variability than spatial prior-based delineation, in both spaces. The Ye and Keren atlases also performed well in specific metrics, such as boundary distance, but no single atlas excelled across all measures. These results highlight the utility of consensus-based and group-level atlases for achieving robust LC segmentation, especially when aiming for anatomical consistency across studies.

These method-dependent differences in LC delineation align with prior work showing that structural and functional localisation approaches achieve overlapping but non-identical LC regions (Mäki-Marttunen and Espeseth, 2021; Turker et al., 2021). Atlas-based labelling produced more spatially coherent LC maps, with principal component analysis indicating tighter clustering and clearer separation along anatomical axes compared to the more diffuse spatial prior-based method, particularly along the left–right and rostro-caudal axes. The superior performance of atlas-based methods may partly reflect acquisition geometry bias. The anisotropic voxels used here may inherently favour the fixed boundaries of atlas-based approaches over spatial prior-based classification, which is more susceptible to partial volume effects in lower-resolution or anisotropic data (Ye et al., 2021). The larger volume of spatial prior–based delineations likely reflects thresholding uncertainty in probabilistic maps, which penalises tissue classification under binary evaluation metrics (Trutti et al., 2021). Despite overall performance differences, the lack of significant anterior–posterior variation indicates partial anatomical convergence across approaches suggesting atlas-based labelling for precision-critical applications and tissue classification where uncertainty modelling is required.

Although individual atlases showed generally high overlap metrics, boundary variability was common across metrics. The inconsistencies likely reflect variation in atlas creation methodology and demographic characteristics (Aganj et al., 2024), which shaped the character of the resulting maps: manual labelling atlases generated deterministic, fixed boundary maps, whereas semi-automated intensity-based approaches produced smoother probabilistic outputs. Population heterogeneity across atlases added further variability. As neuromelanin accumulates with age, younger individuals exhibit lower LC contrast, potentially reducing delineation sensitivity and introducing bias into atlas construction (Berger et al., 2023; Ohm et al., 1997). Most atlases used contrast or signal-to-noise ratio for LC identification, but only two accounted for age-related effects, and one used lower-resolution imaging. Ultra-high-field 7 T MRI imaging markedly improves LC contrast-to-noise and reduces partial-volume effects compared with conventional 3 T sequences, allowing for more reliable volumetric estimates, especially in older adults (Tona et al., 2019). Consequently, the baseline spatial resolution of the source datasets represents a critical methodological confound that dictates downstream delineation outcomes via unavoidable resampling constraints. For a structure with a cross-sectional diameter as small as the LC, the boundary smoothing introduced by resampling to a common target space (see Methods 2.3) artificially expands its effective volume profile and lowers binary overlap metrics such as the Dice coefficient. This explains the modest absolute spatial concordance observed across published atlases. This underscores that baseline acquisition resolution should be carefully considered when interpreting differences across atlases.

In structures as small as the LC, minor boundary mismatches carry disproportional weight on Dice scores; majority-vote consensus references (Ye et al., 2021) can mitigate individual labelling bias and improve cross-atlas comparisons. Aggregating across multiple raters smooths idiosyncratic differences and emphasises consistently labelled regions, even when individual maps are sparse. The result is a more generalisable, anatomically coherent LC representation capturing shared structural features across individuals.

Registration into MNI space improved spatial correspondence predominantly in the central LC, in line with its stronger signal and lower inter-individual variability. Aging-, depression-, and Alzheimer's disease-related changes also concentrate in central or rostral-middle regions (Bae et al., 2025; Hary et al., 2025; Shibata et al., 2007), partly accounting for atlas discrepancies. Additional coverage differences – caudal underrepresentation in the Dahl and Betts atlases, reduced rostral extent in the Liu atlas (Liu et al., 2019) further highlight the need to consider intra-LC variability when evaluating delineation performance.

The biological utility of the atlas-based approach was further demonstrated in the secondary analysis of aging trajectories. Using the atlas-derived LC region in quantitative maps, quadratic regression models identified a significant temporal dissociation between microstructural and macrostructural markers. Specifically, MTsat, a proxy for myelin content (Callaghan et al., 2014), and R1, reflecting longitudinal relaxation rates and shows a correlation with myelin and iron, demonstrated a continuous decline from early adulthood. Conversely, LC volume increased until an inflection point at approximately 54 years, followed by accelerated decline. These results offer a more nuanced perspective to the previously reported diverse results between LC-contrast ratio and age, like no significant correlation in elderly cohorts (Giorgi et al., 2022), an “inverted-U" relationship near the fifth decade of life (Shibata et al., 2006) or a stable plateau in LC contrast after age 57 (Liu et al., 2019). Critically, our use of model-derived effect sizes (*eff*) allowed us to quantify the strength of these relationships more accurately than simple linear correlations. For instance, while LC volume exhibited a negligible linear correlation (r ≈ 0.00), the quadratic model revealed a significant age effect (*eff* = 0.12), confirming that the observed structural stability in early adulthood is a distinct phase of a dynamic nonlinear process. Our findings suggest that while the physical “scaffold” of the LC may remain structurally stable until the mid-50s, consistent with ex-vivo evidence of preserved neuron counts during normal aging (Theofilas et al., 2017), the underlying biochemical composition begins to deteriorate much earlier. This “micro-before-macro” attrition aligns with the hypothesis that compositional shifts, such as neuromelanin‑iron changes or LC shrinkage without cell loss (Theofilas et al., 2017), precede macroscopic volumetric decline. This interpretation is supported by established biophysical models where MTsat and R1 act as “leading indicators” of tissue microstructure, notably, the parallel decline in both metrics reinforces the observation of decreasing macromolecular density (Callaghan et al., 2015). This is reflected in the comparable effect sizes for MTsat and R1 (*eff* = −0.12 to −0.15), suggesting a synchronized deterioration of the LC's microstructure. Furthermore, the continuous increase in PD* likely reflects expanding extracellular space and increased free water content associated with tissue rarefaction. Notably, the lack of significant R2* change in our cohort aligns with reports of heterogeneous or less pronounced iron accumulation in the LC compared to other catecholaminergic nuclei like the substantia nigra (Betts et al., 2019), although a previous study reported a non-significant correlation of −0.09 in R2* with age (Wearn et al., 2024), this lack of association is further confirmed by our minimal R2* effect sizes (*eff* ≤ 0.04).

The observed spatial variability in native space reinforces the importance of using optimized registration algorithms for small nuclei. The high variability and number of outliers, especially in rostral and caudal parts of the LC, highlight the limits of manual labelling and the need for harmonised references. This aligns with previous work supporting group-level LC atlases, tailored to the characteristics of the study cohort, showing greater localisation precision compared to atlases derived from independent populations (Mäki-Marttunen and Espeseth, 2021). Need for harmonization is further supported by recent efforts to aggregate across multiple published LC maps to derive high-confidence meta-masks; by using label fusion and majority-voting to prune low-agreement voxels, these studies have successfully isolated a LC core that mitigates the inherent disagreements in dimensions and localization across individual publications (Dahl et al., 2022; Ye et al., 2021). Altogether, these findings emphasise the value of adopting context-sensitive approaches.

Several limitations should be considered when interpreting findings of this study. Although manual labelling is often treated as ground truth in LC imaging (Doppler et al., 2021; Mäki-Marttunen and Espeseth, 2021) or as correction after automated segmentation (Wolters et al., 2023), our results highlight its inherent variability and limited spatial consistency with Dice scores based on comparisons between manual and automated methods–below 0.4, particularly at the rostral and caudal ends. Inter-operator discrepancies (Daboul et al., 2018) and anatomical ambiguity aggravated by low contrast in neuromelanin-sensitive MRI may have introduced noise into the manual reference labelling, potentially affecting all comparative evaluations. The small sample size (*n* = 24) in this study may limit the generalisability of the results and the robustness of some comparisons. Moreover, the representativeness of the sample in terms of socio-demographic characteristics was not systematically assessed, and the robustness of findings across different scanners or patient groups with varying disease profiles remains to be established. Lastly, we acknowledge the lack of direct post-mortem histopathological validation in our cohort as a relative limitation regarding absolute anatomical specificity. Consequently, our macrostructural and microstructural metrics cannot completely rule out partial volume contamination from adjacent brainstem structures. Similarly, our reliance on 3 T imaging poses spatial resolution constraints compared to emerging ultra-high-field 7 T protocols. Nonetheless, high-throughput in vivo imaging remains a necessary methodology to chart continuous, population-level aging trajectories across thousands of living individuals.

Our study achieved a mean inter-rater reliability of 0.7, aligning with previously reported values in the literature (54–80%) (Betts et al., 2019; Liu et al., 2017; Tona et al., 2017). Second, while we used down-sampled versions of available LC atlases to maintain consistency, this may have reduced the granularity of finer LC features present in the original high-resolution templates. Third, the consensus reference atlas required voxel-wise binarization, which assumes equal atlas quality and may underrepresent high-confidence voxels from individual atlases. This approach, though practical, assumes equal quality and anatomical accuracy across all input atlases and may underrepresent LC voxels with high confidence from single high-quality sources. Lastly, we did not evaluate deep learning-based approaches, which are increasingly used in LC delineation. Recent deep-learning segmentation has achieved high Dice scores, offering a data-driven alternative to atlas-based approaches (Dünnwald et al., 2025). Although outside the scope of this study, such methods offer a promising direction for future work, particularly in large-scale or clinical applications.

We conclude that LC identification in structural MRI remains a complex task, requiring a careful balance between delineation approach, atlas selection and validation strategy. Manual labelling remains a common reference in smaller cohorts, yet the inherent variability and limited reproducibility challenge its role as a definitive ground truth. Automated and atlas-based approaches offer significantly greater scalability when benchmarked against a consensus reference atlas. Our study highlights the relative advantages of atlas-based labelling for achieving spatial precision independent of MRI contrast, allowing for the development of reproducible LC imaging biomarkers in future studies. Probabilistic LC priors provide flexibility in representing anatomical uncertainty when neuromelanin-sensitive imaging is used. Among the eight atlases evaluated, the manually based group-level LC atlas demonstrated robust and generalisable performance across selected statistical metrics and delineation approaches. Modelling trajectories further underscores the value of this approach, revealing a distinct temporal pattern where microstructural decline in MTsat and R1 precedes macroscopic volumetric decline, the latter of which becomes statistically evident only after the fifth decade of life. Our results support atlas-based labelling as a reliable and anatomically consistent approach for LC identification, particularly in combination with harmonised consensus-based reference frameworks. Finally, these findings emphasise the importance of method-appropriate validation metrics and the ongoing need for standardisation in quantitative and neuromelanin-sensitive imaging.

## 5 Data and code availability

The data from the CoLaus|PsyCoLaus cohort used in this study cannot be fully shared due to the inclusion of potentially sensitive patient information. According to the competent authority, the Research Ethic Committee of the Canton of Vaud, Switzerland, sharing or transferring this data would violate Swiss legislation designed to protect participants' personal rights. However, non-identifiable, individual-level data can be made available to researchers who meet the criteria for accessing confidential data, for detailed instructions please see https://www.colaus-psycolaus.ch/professionals/how-to-collaborate/) by CoLaus|PsyCoLaus Datacenter (CHUV, Lausanne, Switzerland). Code to all analyses and figures will be uploaded to https://github.com/dmiolga/LC_delineation.git once the paper has been accepted.

## CRediT authorship contribution statement

**Olga Dmitrichenko:** Writing – review & editing, Writing – original draft, Visualization, Validation, Methodology, Investigation, Formal analysis, Conceptualization. **Giuseppina Baldizzi:** Writing – review & editing, Writing – original draft, Visualization, Validation, Methodology, Formal analysis, Data curation. **Tatjana Schmidt:** Writing – review & editing, Data curation. **Aurélie Bussy:** Writing – review & editing, Methodology. **Olivier Colliot:** Writing – review & editing, Methodology. **Antoine Lutti:** Writing – review & editing, Resources, Data curation. **Ferath Kherif:** Writing – review & editing, Resources, Methodology. **Bogdan Draganski:** Writing – review & editing, Writing – original draft, Supervision, Resources, Project administration, Methodology, Investigation, Funding acquisition, Data curation, Conceptualization.

## Acknowledgments and funding

B.D. is supported by the Swiss National Science Foundation (project grant no. 213595, 32003B_135679, 32003B_159780, 324730_192755 and CRSK-3_190185), ERA_NET NEURON JTC2020: iSEE and JTC2023-ELSA: BrainTree projects and the InnoSuisse Flagship Swiss brAInHealth project. O.C. has received funding from the French government under management of Agence Nationale de la Recherche as part of the “France 2030” program (reference ANR-23-IACL-0008, project PRAIRIE-PSAI), as part of the “Investissements d'avenir” program (reference ANR-19-P3IA-0001, project PRAIRIE 3IA Institute and reference ANR-10-IAIHU-06, project Agence Nationale de la Recherche-10-IA Institut Hospitalo-Universitaire-6) and from the European Union's Horizon Europe Framework Programme (grant number 101136607, project CLARA). A.L. is supported by the 10.13039/501100001711Swiss National Science Foundation (grant no. 320030_184784, CR00I5-235940). The Laboratory for Research in Neuroimaging-LREN is very grateful to the Roger De Spoelberch and Partridge Foundations for their generous financial support.

## Declaration of competing interest

Other authors declare that they have no known competing financial interests or personal relationships that could have appeared to influence the work reported in this paper.

## Data Availability

Data will be made available on request.
